# Image cytometric analysis of p53 and mdm-2 expression in primary and recurrent mucoepidermoid carcinoma of parotid gland: immunohistochemical study

**DOI:** 10.1186/1746-1596-5-72

**Published:** 2010-11-22

**Authors:** Ehab S Abd-Elhamid, Mohamed H Elmalahy

**Affiliations:** 1 Associate Professor, Oral Pathology Department, Faculty of Dentistry, Ain Shams University, Cairo, Egypt; 2 Associate Professor, Oral Pathology Department, Faculty of Oral and Dental Medicine, Elmenia University, Cairo, Egypt

## Abstract

**Aims and Objectives:**

This study aims to analyze immunocytochemically p53 aberrant expression and mdm-2 expression in primary and recurrent mucoepidermoid carcinoma (MEC) of parotid gland and to ascertain if expression of these markers correlates with tumor behavior, clinical outcome, histological grade and local recurrence.

**Methods:**

20 cases histologically diagnosed as primary MEC with different grades were included in the study. Out of 20 cases, 7 were classified as grade I, 8 as grade II and 5 as grade III. Immunohistochemical staining of these 20 primary cases as well as 6 recurrent cases with anti-p53 and anti-mdm-2 antibodies was carried out. Area fraction of immunopositivity was estimated by image analysis software.

**Results:**

16/20 primary cases were p53 +ve (80%). The p53 positive cases included 3 cases classified as grade (I), 8 cases as grade (II) and 5 cases as grade (III). All 6 recurrent cases were p53 +ve. On the other hand, 14/20 primary and only 2/6 recurrent cases were mdm-2 +ve. The mdm-2 +ve primary cases included 2 classified as grade (I), 7 as grade (II) and 5 as grade (III). 12 primary MEC showed co-expression of both p53 and mdm-2 of which 2 cases showed local recurrence.

**Conclusions:**

these data suggested that expression of p53 and mdm-2 in primary and recurrent MEC correlates with the high histological grade. P53 aberrant expression is not only considered as an early event in MEC carcinogenesis but also correlates to tumor behavior and local recurrence. Mdm-2 overexpression is correlated to pathogenesis of MEC. However, no strong evidence was found between mdm-2 expression and MEC local recurrence.

## Background

Malignant tumors of the major salivary glands are rare, representing 7% of all head and neck cancers. Benign and malignant parotid gland neoplasms account for approximately 90% of all major salivary tumors. They are characterized by a variety of histologic subtypes with different biologic and prognostic behavior [1].

Recent studies of the molecular biology of cancers have demonstrated that the loss of function of tumor suppressor genes may elicit tumorigenesis, and may be associated with the development and progression of many different cancer types. One of the best known tumor suppressor genes is the p53 gene [2]. This gene is located on human chromosome 17(p13) and encodes a nuclear phosphoprotein (53 Kd) that thought to regulate proliferation of normal cells [2]. Various point mutations of the p53 gene usually determine the transcription of a mutated protein, which is much more metabolically stable than the wild protein and accumulates in the nucleus. Consequently, normal cells with the wild p53 gene do not show p53 immunoreactivity, whereas tumors with the mutant form of the p53 gene may express high levels of p53 protein that are immunohistochemically detectable. The accumulation of the p53 oncoprotein clearly has been shown to correlate with shorter survival in patients with carcinomas of the breast, lung, stomach, colon as well as bladder [3].

The mdm-2 oncogene was first cloned as an amplified gene on a murine double-minute chromosome in the 3T3DM cell line, a spontaneously transformed derivative of BALByc 3T3 cells [4]. The gene encodes a 489 amino acid polypeptide that contains a p53 binding domain, an acidic region, and three putative zinc-binding motifs (one zinc-finger and one RING finger). Overexpression of the mdm-2 gene in NIH 3T3 cells increases the tumorigenic potential of these cells, thus establishing mdm-2 as an oncogene [4]. The mdm-2 gene can immortalize rat embryo fibroblasts and cooperate with the activated ras oncogene to transform these cells [5]. The mdm-2 gene is amplified or overexpressed in about 40-60% of human osteogenic sarcomas and about 30% of soft tissue sarcomas [4,5], implicating its role in the development of these malignancies.

An important function of mdm-2 is to bind to the p53 tumor suppressor protein, inhibiting its ability to act as a transcription factor [6]. P53 also activates mdm-2 expression at the level of transcription suggesting that mdm-2 can function as a negative feedback regulator of p53 [7].

Several studies were carried out to investigate the role of aberrant expression of p53 tumor suppressor gene and mdm-2 overexpression in different benign and malignant salivary gland tumors [8,9]. However, no definite data was available in the literature regarding the exact role of p53 and mdm-2 mutation upon the clinical coutcome of muco-epidermoid carcinoma as one of the common malignant salivary gland tumors and also the factors that might be related to the aggressiveness and recurrence of these tumors.

The aim of this study is to analyze immunocytochemically p53 aberrant expression and mdm-2 expression in primary and recurrent MEC of parotid gland and to ascertain if expression of these markers correlates with tumor behavior, clinical outcome, histological grade and local recurrence.

## Method

### Case selection

Twenty cases of primary muco-epidermoid carcinoma of the parotid gland collected from June 1998 through December 2004 were obtained from the files of the Pathology Department, National Cancer Institute, Cairo University (Cairo, Egypt). Clinical information was obtained from the patients' clinical records. Clinical staging was assayed by using the International Union Against Cancer (1987) TNM staging system [10].

All of the patients with parotid masses were surgically treated with total parotidectomy. Facial nerve preservation was performed in 14 (70%) of the cases. A facial nerve weakness by tumor infiltration in 12 (60%) and a close tumor adherence to the main trunk or to one of the main division branches of the facial nerve justified its partial or total sacrifice in 6 (30%) of the cases. Total parotidectomy was performed in association with homolateral radical neck dissection in six (N1) cases and with functional neck dissection in five (N0) cases. In addition, 2 patients who did not undergo neck procedures associated with the parotid surgery presented with regional lymph node metastases and underwent radical neck dissection during the follow-up period.

Pathologic examination of the neck specimens confirmed tumor involvement in all patients with clinically palpable cervical lymph nodes.

After primary surgery, 11 of 20 cases (55%), including all patients with cervical lymph node metastases and those with clinical signs of local infiltration of skin, bone, muscle, and facial nerve, received a full course of postoperative radiotherapy to the parotid area and to the neck.

All patients were followed for a minimum of 36 months (mean follow-up, 42 months).

### Tissue samples and immunohistochemistry

Formalin fixed, paraffin embedded specimens of the primary parotid gland tumors from each patient were available for immunohistochemical analysis. Tissue specimens from recurrent cases were also included in the analysis. Immunohistochemistry was performed on deparaffinized, 5-μm sections after antigen retrieval using microwave oven heating.

A murine monoclonal antibody, DO-7 antihuman p53 protein, specific for a formalin-resistant epitope of the N-terminus of the human protein reacting with both wild and mutant types of the p53 protein (Dako, Copenhagen, Denmark) was used.

For immunodetection of mdm-2, anti-mdm-2 mouse IgG-Kappa monoclonal antibody specific for epitope located within the amino acids 26-169 of the human mdm-2 protein (Zymed Laboratories Inc., USA) was used.

For immunostaining, the avidin-biotin-peroxidase complex method was used according to manufacturer instructions. In brief, after deparaffinization and inactivation of endogenous peroxidase activity and blocking of cross reactivity with pre-immune serum (Peroxide Block Kit, Zymed Laboratories Inc., USA), the sections were incubated for 1 hour at room temperature with the primary antibody diluted at 1:50. Localization of the primary antibody was achieved by subsequent incubation of biotinylated anti-primary antibody with an avidin- biotin complex conjugated to horseradish peroxidase, and diaminobenzidine (Zymed Laboratories Inc., USA). The slides were washed three times with phosphate buffered saline after each incubation. Negative controls were performed by substituting the primary antibody with non-immune mouse serum.

### Image Cytometric Analysis

In this study, the immunostaining of each antibody was assessed in 4 serial sections from each specimen. For evaluation of p53 and mdm-2 immunoreactivity, tumor cells were considered immunopositive when they displayed a brownish nuclear and/or cytoplasmic immunoreactivity.

Surface area of the immunopositivity was calculated by using the image analysis software (ImageJ, NIH, 1.31b, USA) and the area fraction of immunopositivity was then estimated by calculating the ratio of surface area of immunopositive cells to the total area of the microscopic field. Data were then collected, tabulated for further statistical assessment of the results.

### Statistical Analysis

ANOVA test followed by Post Hoc multiple comparison test (Tukey HSD method) were performed to compare the mean area fraction of immunopositivity (Nuclear and/or cytoplasmic) of either p53 or mdm-2 in primary and recurrent cases in relation to different grades of MEC. Welch two sample t-test was performed to compare mean area fraction of immunopositivity in primary versus recurrent cases for each antibody separately. Pearson's correlation was done to study the correlation between P53 and mdm-2 immunopositivity in different grades of primary and recurrent MEC.

Statistical analysis for survival rates was not performed as the data for time of mortality after the primary surgery was not available.

## Results and Discussion

After follow-up period of all patients, 6 cases showed evidence of recurrence (30% of cases). The time elapsed between the surgical removal of the primary lesion and the recurrence was variable and ranged from 32-40 months.

### Histopathological Results

**Figure 1:**
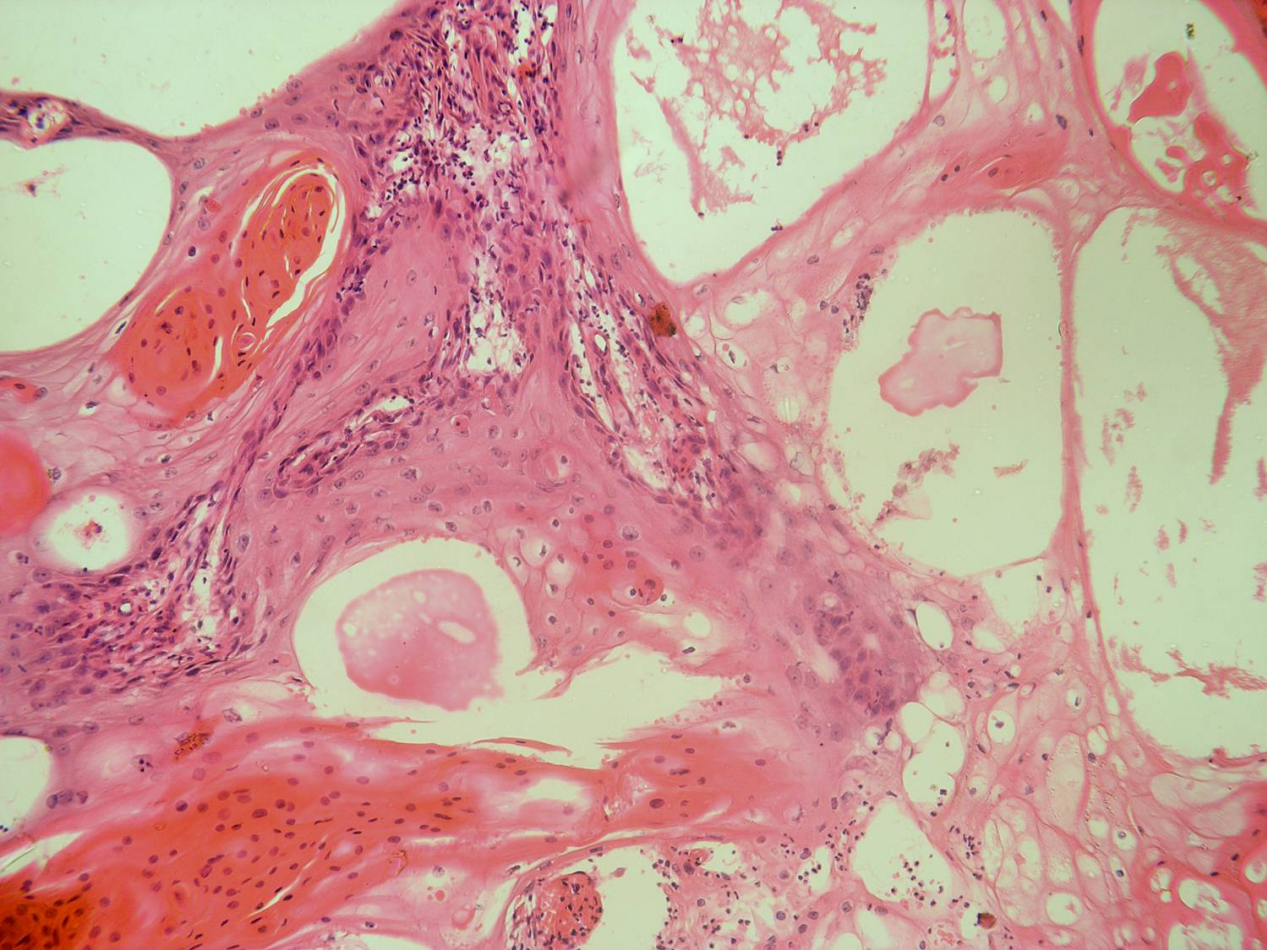
**Low grade MEC showing cystic spaces and duct-like structures lined by mucous-secreting cells together with few differentiated epidermoid cells (HE × 200)**.

**Figure 2:**
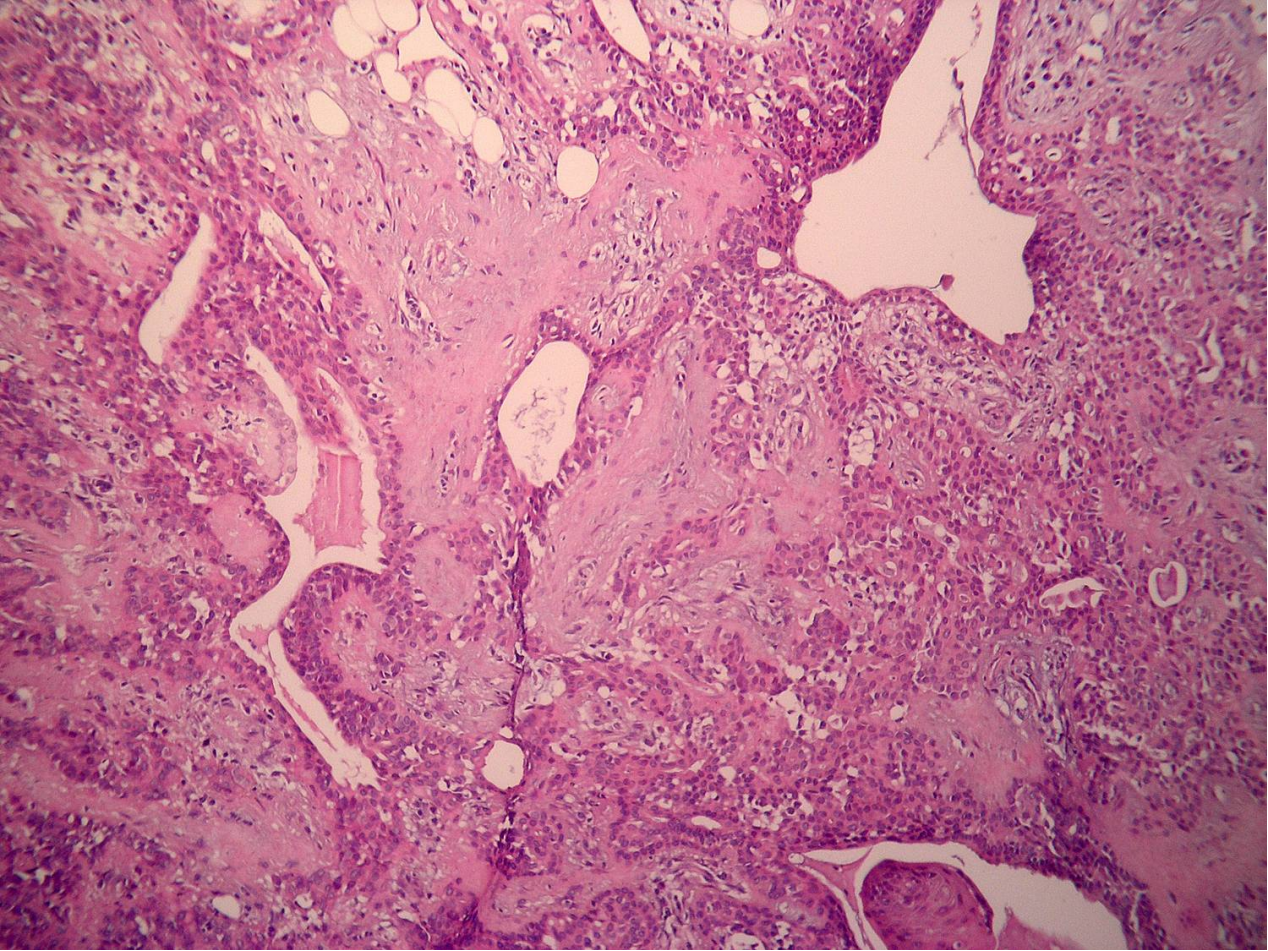
**Intermediate grade MEC showing tumor nests formed of epidermoid cells intermingled with clear mucous cells together with duct-like structures lined by both types of cells (HE × 200)**.

**Figure 3:**
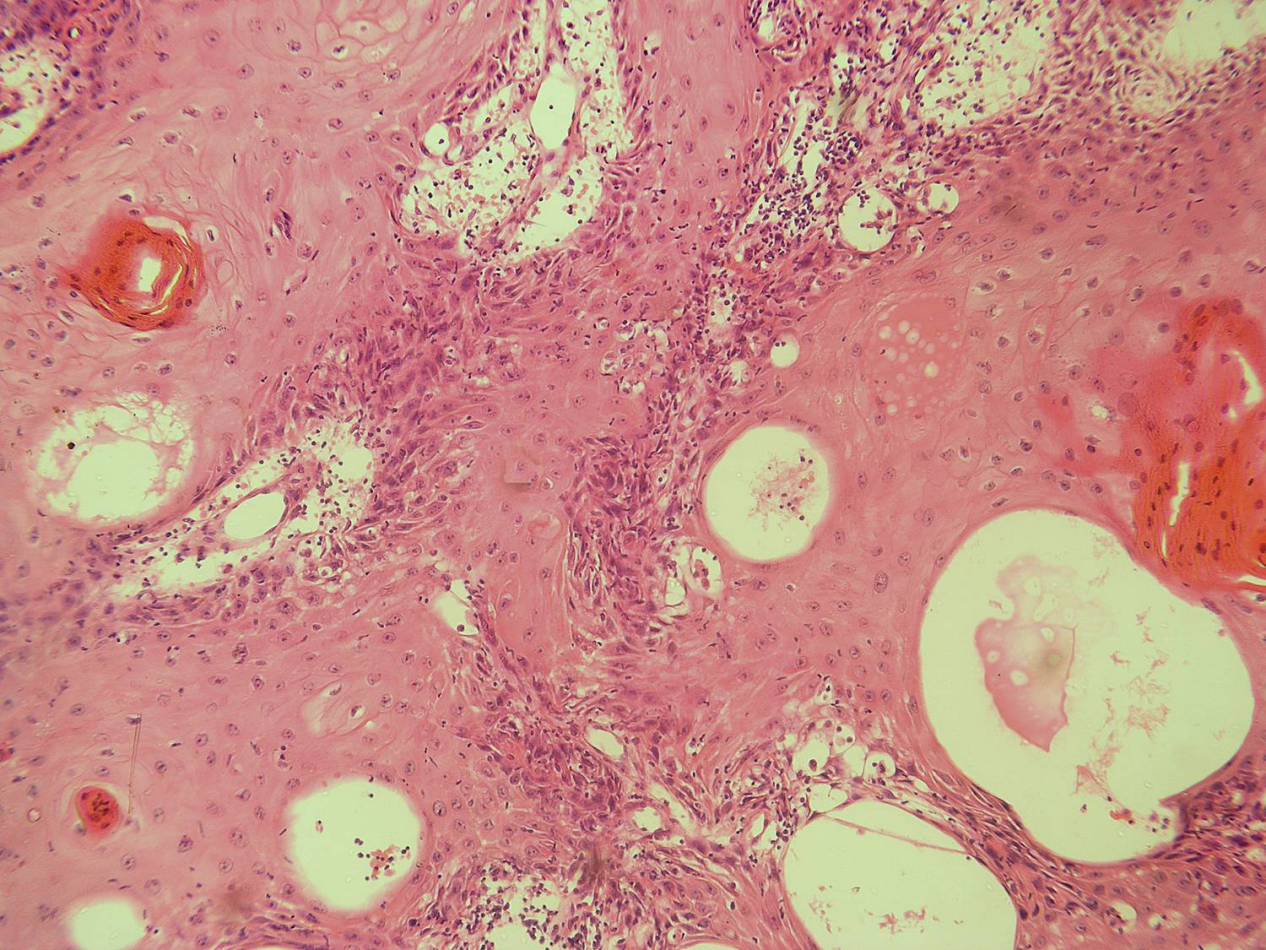
**High grade MEC showing nests of epidermoid cells some of which are differentiated into epithelial pearls with central keratinization**. Note the few scattered mucous-secreting cells (HE × 200).

Out of the twenty cases under study, 7 (35%) were histologically graded as low grade (Grade I) (Figure 1), 8 (40%) were histologically graded as intermediate grade (Grade II) (Figure 2) and 5 (25%) were graded as high grade (Grade III) (Figure 3).Three recurrent lesions were histologically graded as high grade (grade III) while the remaining three lesions were graded as intermediate grade (grade II). Two other patients rather than the six recurrent cases died after surgical removal of the primary tumor due to distant metastasis to the bones.

(Table 1) demonstrated the clinical data of all cases under study and the histopathological grading of lesions.

**Table 1:** Clinicopathologic data of all cases under study

Case	Age	Gender	Histological grade	Facial nerve involvement	Metastasis	Recurrence
1	35	♂	II	1*	N0	0**

2	22	♀	III	1	N1	0

3	15	♂	II	1	N1	1

4	49	♀	II	1	N0	0

5	44	♀	I	0	N0	0

6	33	♀	III	1	N1	1

7	17	♂	II	1	N1	1

8	11	♂	III	1	N1	1

9	24	♀	I	0	N0	0

10	60	♂	II	1	N0	0

11	38	♀	I	0	N0	0

12	46	♀	II	1	N1	1

13	16	♂	II	1	N0	0

14	48	♂	I	0	N0	0

15	51	♂	III	1	N1	1

16	46	♀	III	1	N1	0

17	13	♀	II	0	N0	0

18	57	♂	I	0	N0	0

19	14	♀	I	0	N0	0

*20*	31	♂	I	0	N0	0

* 1 = Yes ** 0 = No

#### P53 Immunohistochemical Results

**Figure 4:**
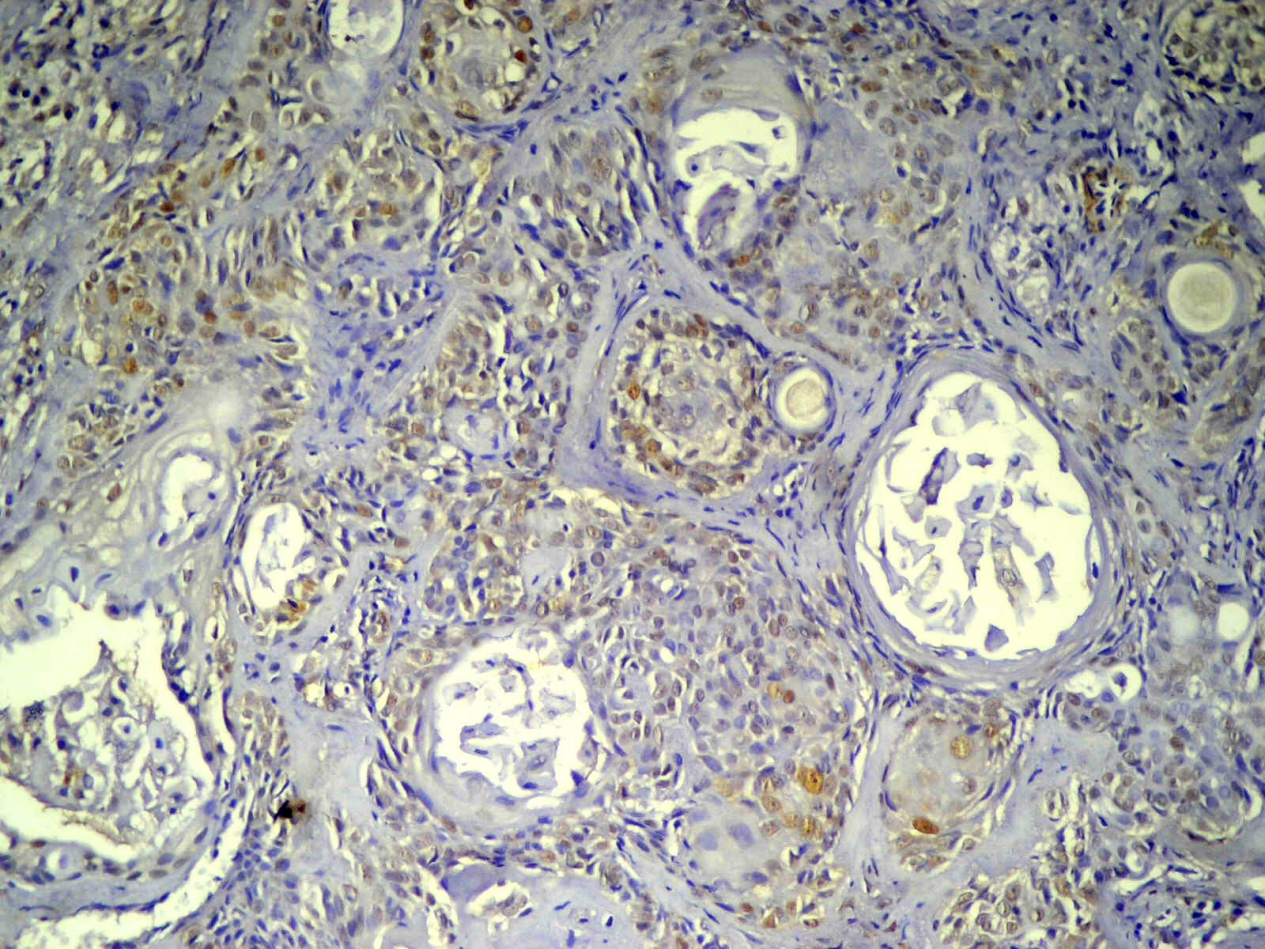
**Primary MEC showing positive nuclear immunostaining of epidermoid cells forming tumor nests**. The mucous cells were immunonegative (Anti-p53 × 100).

Sixteen out of the 20 primary cases under study showed positive immunostaining for p53 (80%). The p53 positive cases included 3 classified as grade (I), 8 as grade (II) and 5 as grade (III) (Table 2). The reaction was nuclear or nuclear/cytoplasmic in localization and granular brownish in nature. The immunopositivity was confined to epidermoid cells that formed cell nests while the vacuolated mucous secreting cells that formed duct-like structures were immunonegative (Figure 4). The positive nests of epidermoid cells showed nuclear staining of most of the peripheral cells with few immunopositivity noted in the central cells.

**Table 2:** Summary of P53 and Mdm-2 immunopositive cases in relation to histological grade and lymph node metastasis.

Histological Grade	P53 immunopositivity				Mdm-2 immunopositivity			
	
	Primary		Recurrent		Primary		Recurrent	
	
	N0	N1	N0	N1	N0	N1	N0	N1
Grade I	3	0	0	0	2	0	0	0

Grade II	5	3	0	3	4	3	0	1

Grade III	2	3	0	3	2	3	0	1

**Total**	**10**	**6**	**0**	**6**	**8**	**6**	**0**	**2**

***Grand Total***	**16**		**6**		**14**		**2**	

**Figure 5:**
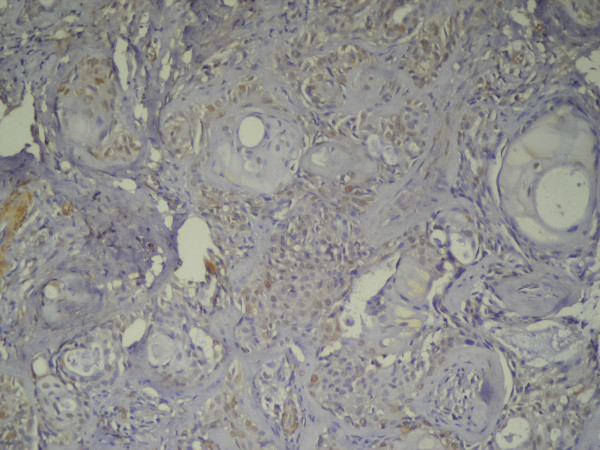
**Recurrent MEC showing positive nuclear immunostaining of epidermoid cells forming the advancing front of the tumor nests**. The mucous cells were immunonegative (Anti-p53 × 100).

The six recurrent cases showed p53 immunopositivity (Table 2). The localization and nature of the reaction was similar to the primary lesions. However, the immunopositivity was confined to the epidermoid cells that formed the advancing front of the nests (Figure 5).

#### Mdm2 Immunohistolochemical Results

**Figure 6:**
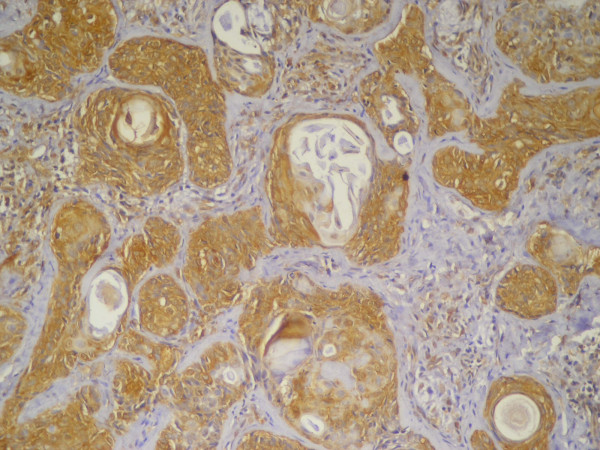
**Primary MEC showing positive nuclear and cytoplasmic immunostaining of epidermoid cells forming tumor nests**. The mucous cells were immunonegative (Anti-mdm-2 × 100).

Fourteen out of the 20 primary lesions (70%) were positively stained with mdm2. The mdm-2-positive cases included 2 lesions classified as grade (I), 7 cases as grade (II) and 5 cases as grade (III) (Table 2). The reaction was brownish and granular in nature and was cytoplasmic in most of positive cells with few cells showed nuclear/cytoplasmic localization of the immunoreaction. The majority of the neoplastic epidermoid cells were immunopositive while the mucous secreting cells were immunonegative. Both the peripheral as well as central cells of the tumor nests showed immunopositive reaction (Figure 6).

**Figure 7:**
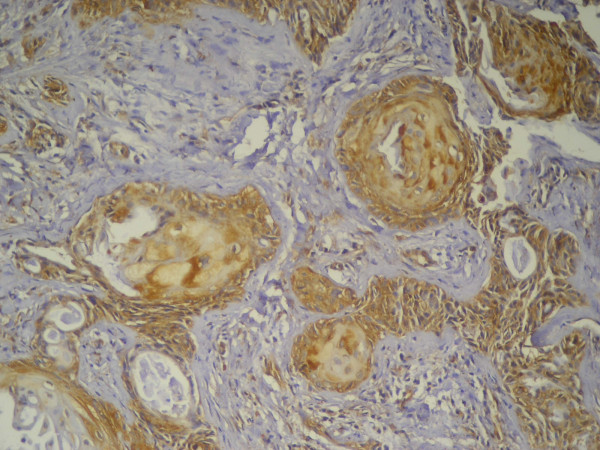
**Recurrent MEC showing positive nuclear and cytoplasmic immunostaining of epidermoid cells forming tumor nests**. The mucous cells were immunonegative (Anti-mdm-2 × 100).

Two lesions only out of the six recurrent cases showed immunopositivity to mdm2 (Table 2). The immunopositivity was noted in epidermoid cells that surrounded the central keratinized cells while most of the peripheral tumor cells as well as mucous secreting cells that formed the duct-like structure were immunonegative (Figure 7).

#### Image Cytometric Results

The mean area fraction of p53 immunopositivity in primary MEC was (23.1%) while the mean area fraction of recurrent cases was (18.2%). On the other hand, the mean area fraction of mdm-2 immunopositivity was (40.9%) and (34.1%) in primary and recurrent MEC respectively.

A summary of the mean area fraction of immunopositivity in both primary and recurrent MEC stained with p53 and mdm-2 is shown in (table 3).

**Table 3:** Summary of mean area fraction of both p53 and mdm-2 immunopositivity in relation to recurrence in different grades of MEC:

Histological Grade	P53 mean area fraction (%)		Mdm-2 mean area fraction (%)	
	
	Primary	Recurrent	Primary	Recurrent
Grade I	13.2	0	33.4	0

Grade II	25.3	16.5	39.9	31.9

Grade III	30.7	19.8	49.3	36.2

***Grand Mean***	**23.1**	**18.2**	**40.9**	**34.1**

#### Statistical Results

ANOVA test and Post Hoc multiple comparisons test revealed a statistically significant increase in the mean area fraction of p53 or mdm-2 immunopositivity from low grade to high grade MEC (Tables 4, 5).

**Table 4:** ANOVA test and Post Hoc multiple comparisons of mean area fraction of p53 immunopositivity in relation to different grades of MEC:

Descriptive statistics (P53)						
**Grade**	**Number of fields (Records)**		**Mean Area Fraction**		**Standard Deviation**	

Grade I	12		13.18917		1.426180	

Grade II	44		20.91700		5.846319	

Grade III	32		25.26250		7.679862	

**ANOVA (p53)**						

P53		**Sum of Squares**	**df**	**Mean Square**	**F**	**P Value**
	
	Between Groups	1295.4	2	647.68	16.478	**0.0001**
		
	Within Groups	3183.8	81	39.31		
		
	Total	686.99	83			

**Post Hoc (Tukey HSD)**						

**Dependent Variable**	**(I) Groups**	**(J) Groups**	**Mean Diff**.	**P Value**		**Significance**

P53 Mean Area Fraction	Grade I	Grade II	-7.727833	**< 0.001**		S*
		
		Grade III	-12.073333	**< 0.001**		S
	
	Grade II	Grade III	- 4.345500	**< 0.001**		S

* The mean difference is significant at the 0.05 level

**Table 5:** ANOVA test and Post Hoc multiple comparisons of mean area fraction of mdm-2 immunopositivity in relation to different grades of MEC:

Descriptive statistics (mdm-2)						
**Grade**	**Number of fields (Records)**		**Mean Area Fraction**		**Standard Deviation**	

Grade I	8		32.16375		1.264538	

Grade II	32		35.95156		7.165884	

Grade III	24		42.77000		5.654459	

**ANOVA (mdm-2)**						

		**Sum of Squares**	**df**	**Mean Square**	**F**	**P Value**
	
Mdm-2	Between Groups	952.76	2	476.38	12.427	
		
	Within Groups	2338.42	61	38.33		0.0001
		
	Total	3291.18	63			

**Post Hoc (Tukey HSD)**						

**Dependent Variable**		**(I) Groups**	**(J) Groups**.	**Mean Diff**	**P Value**	**Significance**

Mdm-2 Mean Area Fraction		Grade I	Grade II	-3.787812	< 0.001	S*
			
			Grade III	-10.606250	< 0.001	S
		
		Grade II	Grade III	- 6.818438	> 0.05	NS

* The mean difference is significant at the 0.05 level

Welch two samples t-test showed a statistically significant decrease in the expression of either p53 or mdm-2 from primary to recurrent cases regardless of the tumor grade (Table 6).

**Table 6:** Welch two samples t-test for comparison of mean area fraction (AF) of p53 and mdm-2 immunopositivity in primary versus recurrent MEC:

Welch Two Sample t-test (P53)							
Dependent Variable	MEC	Mean AF	95% Confidence Interval		t	df	P value
						
			Lower Bound	Upper Bound			
P53 Mean Area Fraction	**Primary MEC**	23.12033	4.042945	9.200222	5.1179	72.87	0.00001
						
	Recurrent MEC	**18.23875**					

**Welch Two Sample t-test (Mdm-2)**							

**Dependent Variable**	**MEC**	**Mean AF**	**95% Confidence Interval**		**t**	**df**	**P value**
						
			**Lower Bound**	**Upper Bound**			

Mdm-2 Mean Area Fraction	**Primary MEC**	40.89893	6.800357	11.822500	7.5658	30.697	0.00001
						
	**Recurrent MEC**	34.14750					

* The mean difference is significant at the 0.05 level

**Figure 8:**
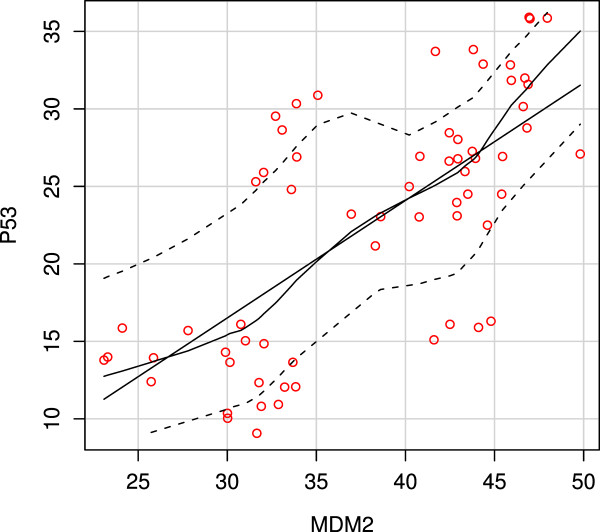
**A scatter plot representing a linear positive correlation between p53 and mdm-2 expression in MEC**.

On the other hand, regression analysis revealed a linear positive correlation between the expression of p53 and mdm-2 in relation to either tumor grade in both primary and recurrent MEC (Figure 8). However, this correlation was not proved to be existed when comparing the expression of both markers in recurrent cases only (Table 7).

**Table 7:** Correlation with regression analysis of p53 and mdm-2 immunopositivity in primary and/or recurrent MEC

Pearson's Correlation (in both primary and recurrent cases)			
		**Mean AF-p53**	**Mean AF-Mdm-2**

**Mean AF-p53**	**Pearson Correlation**	1	0.4762 (**)
	
	**Sig. (2-tailed)**		0.0001

**Mean AF-Mdm-2**	**R Squared**	0.4762 (**)	1
	
	**Sig. (2-tailed)**	0.0001	

**Pearson's Correlation (in primary cases only)**			
		**Mean AF-p53**	**Mean AF-Mdm-2**

**Mean AF-p53**	**Pearson Correlation**	1	0.3864 (**)
	
	**Sig. (2-tailed)**		0.0001

**Mean AF-Mdm-2**	**R Squared**	0.3864 (**)	1
	
	**Sig. (2-tailed)**	0.0001	

**Pearson's Correlation (in recurrent cases only)**			

		**Mean AF-p53**	**Mean AF-Mdm-2**

**Mean AF-p53**	**Pearson Correlation**	1	-0.08434
	
	**Sig. (2-tailed)**		0.5249

**Mean AF-Mdm-2**	**R Squared**	-0.08434	1
	
	**Sig. (2-tailed)**	0.5249	

** Correlation is significant at the 0.01 level (2-tailed).

## Discussion

This aim of the present study was to investigate the immunoexpression of p53 and mdm2 oncoproteins in primary and recurrent MEC as a trial to clarify the possible role of those proteins in the pathogenesis of this tumor and also to correlate the effect of their expression on the recurrence and clinical outcome of these tumors of salivary glands.

Earlier studies focused on the analysis of p53 gene in tumors of the salivary glands; however the connection between salivary gland carcinogenesis and the mdm2 oncoprotein was still elusive [11]. Some authors demonstrated that not only p53 gene is a potential target in neoplastic transformation, but also genes involved in the regulation of its function since mdm2 can potently regulate p53, and functions as an oncogene in the process of cell transformation [12]. For this reason co-expression of both p53 and mdm2 was investigated for their role in the biological behavior of MEC.

Interpretation of immunohistochemical positivity as mild, moderate and intense reaction is now considered as an obsolete method as it lacks the basic standardized parameters upon which the results are interpreted and also due to difficulty to avoid the subjectivity in estimating the degree of immunostaining. For this reason, a novel method of image analysis by which the results were automatically estimated as a surface area of immunopositivity is widely used [13].

Many authors considered both nuclear and cytoplasmic staining or exclusively cytoplasmic staining as positive results [14], and this was chosen for assessment of immunopositive reaction of mdm2 in this study, because a strong evidence is now available that mdm2 is an RNA binding protein that can shuttle between the nucleus and the cytoplasm, and both mdm2 and p53 proteins contain a nuclear-import and nuclear-export signals (NES) that enable them to be directed into the nucleus and out again towards the cytoplasm [14]. This NES of mdm2 is essential for p53 degradation by interacting with cytoplasmic proteasomes, where p53 is specifically degraded [15].

Immunohistochemical results of the present study revealed that 80% of primary lesions showed immunopositive expression of p53. These results were in accordance with that found by many authors who reported an immunopositive expression of aberrant p53 in glandular carcinomas including those of salivary glands and considered expression of mutant p53 as an early event in malignant transformation of these tissues [9,16-18]. Nordkvist et al [19] and Ohki et al [20] have detected p53 gene alterations in some MEC lesions. Although a small number of MEC cases were analyzed in these studies, p53 gene alterations were identified, suggesting a possible involvement of this gene in MEC pathogenesis. Matizonkas-Antontio et al [1] utilizing single-stranded conformational polymorphism (SSCP) analysis for p53 mutations in salivary gland tumors confirmed these data and reported that p53 mutations in exons 5 and 8 were most likely related to salivary gland neoplasms. They added that mutations were observed in 1 of 3 of MEC cases under study. On the other hand, Karja et al [18] did not observe any p53 mutations in these lesions suggesting variable results regarding p53 gene status in these lesions.

P53 immunopositivity of epidermoid cells observed in the present study might indicate the proliferative nature of these cells when compared to a more differentiated mucous-secreting cells. These results together with that of mean area fraction of immunopositivity seen by image analysis suggested a more expression of p53 in these aggressive cell population in MEC indicating a possible relationship between alteration of p53 function and malignant transformation of these cells [18]. This suggestion might explain the immunopositivity of p53 in all intermediate and high grade cases containing predominantly a more aggressive epidermoid and intermediate cells while only 3 out of 7 cases classified as grade (I) showed p53 immunopositivity due to presence of a more differentiated and less aggressive mucous-secreting cells in these lesions.

Immunopositivity of p53 in all recurrent cases noted in the present study strongly suggested the argument of Oreste Gallo et al [8] who stated that p53 expression tended to be higher in late stage cancers, correlated with clinicopathologic variables indicative of aggressiveness, such as regional and distant metastases; and provided prognostic information for disease free and overall survival probabilities. In fact, the above-mentioned trends found in p53-positive parotid gland cancers indicated that p53 gene expression does influence tumor behavior and strongly suggested that parotid gland carcinomas showing high p53 oncoprotein immunoreactivity are aggressive and have a poor prognosis as already was observed for other glandular carcinomas such as lung, breast, stomach, and colon cancer [3].

Mdm-2 immunohistochemical results of the present study revealed that 70% of MEC showed mdm-2 immunopositivity. These data agreed with those reported by Haitel et al [21] and Higashiyama et al [22] who reported an overexpression of mdm-2 oncoprotein in clear cell renal carcinoma and non-small cell lung cancer respectively.

However, de Araujo et al [9] found that the expression of mdm-2 in malignant tumors of minor salivary glands is not significantly high when compared to that in benign tumors.

On the other hand, only 2 out of the six recurrent cases showed mdm-2 immunopositivity. These data suggested that expression of mdm-2 is an early event in the carcinogenic pathway of MEC and hence the lowered percentage of mdm-2 immunopositivity is usually related to tumors of better prognosis and/or low recurrence rate [23].

The data of the present study also revealed that out of the 16 p53 immunopositive primary cases, 12 were mdm-2- positive while only 4 cases were p53 +ve/mdm-2 -ve. The high percentage of primary MEC that showed co-expression of both p53 and mdm-2 together with the linear correlation noted by regression analysis might indicate the possible interaction between these two proteins. One possible scenario of this interaction is that p53 aberrant expression as an early event in the carcinogenic cascade of MEC led to activation of mdm-2 feedback loop for proteasome degradation of activated p53 [14]. Despite the activation of mdm-2, degradation of p53 do not takes place. One explanation of this argument is that mdm-2 had two apparently opposite functions, a tumorigenic function and a growth arrest one. This dual role of mdm-2 could depend on the level of mdm-2 expressed in the cell and also the balance of positive and negative regulators of cell cycle which is critical for the control of cell proliferation [24].

Another explanation is that phosphorylation of the amino terminus of p53 does not affect its DNA binding ability, but does affect its affinity for mdm-2 and subsequent p53 degradation [25]. So, overexpression of p53 would increase the levels of mdm-2 by the feed back loop but without affecting the activity of phosphorylated p53 [25].

These explanations on the immunohistochemical level would focus on the role of these two proteins in the pathogenesis and clinical outcome of MEC, however, further studies utilizing much more advanced research tools such as in situ hybridization and SSCP analysis are highly recommended.

## Conclusions

Based upon the results of the present study, it could be concluded that:

• Expression of p53 and mdm-2 in primary and recurrent MEC correlates with the high histological grade.

• P53 aberrant expression is not only considered as an early event in MEC carcinogenesis but also correlates to tumor behavior and local recurrence.

• Mdm-2 overexpression is correlated to pathogenesis of MEC. However, no strong evidence was found between mdm-2 expression and MEC local recurrence.

## Competing interests

The authors declare that they have no competing interests.

## Authors' contributions

**E.S.A **Participated in the study design, collection of the background references, photomicrography of the immunohistochemical results, interpreting and displaying the results of the study, writing the discussion of the results, alignment of the references, carried out the sequence alignment and drafted the manuscript.

**M.H.E **participated in the study design, collection of the background references, carried out the immunohistochemical technique, participated in displaying the results of the study, writing the discussion of the results and alignment of the references
